# Post-traumatic stress disorder in parents of schizophrenic patients at the Arrazi Psychiatric Hospital in Salé following familial violence

**DOI:** 10.1192/j.eurpsy.2024.1380

**Published:** 2024-08-27

**Authors:** N. Ait Bensaid, F. El Omari

**Affiliations:** Arrazi psychiatric hospital in salé, Salé, Morocco

## Abstract

**Introduction:**

Parents whose adult child has a serious mental illness are at risk of serious violence from their child. One of the reasons for the high risk of PTSD is violence in the home [1,2]. The high risk of PTSD in parents of patients with schizophrenia is an issue of great concern because parents are likely to fear repeated violence and, therefore, to object to patients being discharged from hospital.

**Objectives:**

To assess the existence of post-traumatic stress disorder in relatives of patients with schizophrenia treated at the Arrazi University Psychiatric Hospital in Salé following familial violence.

**Methods:**

This was a descriptive cross-sectional study using a questionnaire including sociodemographic criteria, clinical criteria, questions about domestic violence and an “IES-R” post-traumatic stress symptom assessment questionnaire to investigate the existence of post-traumatic stress disorder in relatives of patients with schizophrenia followed up at Arrazi University Psychiatric Hospital in Salé following familial violence.

**Results:**

The response of 72 relatives of schizophrenic patients was collected. About 70% of the participants were mothers. The average age of the participants was 58. All lived with children who had been treated for schizophrenia for more than 18 years (57% of participants). Around 20% of these children were in hospital at the time of completing this questionnaire.

About 80% of the children with schizophrenia spent all their time at home, and about 89% of the participants had already been victims of violence from their sick children. 90% had been sworn at and insulted, the majority blamed themselves for the illness, about 56% had already been kicked or punched, and 36% had already received death threats and 12% serious injuries/.

For all items, parents with a high IES-R score had significantly more experiences of violence than parents with a low IES-R score. The percentage of parents with a high IES-R score was 45%.

**Conclusions:**

The experience of severe violence and hospitalisation of a patient was related to a high risk of post-traumatic stress disorder in parents. These two factors can be considered as traumatic events arising from crisis situations and can have harmful consequences for parents and their schizophrenic children, who are sometimes rejected. There seems to be a need to create crisis intervention programmes that offer a multidisciplinary approach capable of rapidly detecting the exacerbation of a serious mental illness and providing rapid and intensive treatment as quickly as possible. Finally, the provision of support, education or treatment for parents during their child’s hospitalisation is essential.

**Disclosure of Interest:**

None Declared

